# Effects of Anti-Parkinsonian Drugs on Verbal Fluency in Patients with Parkinson’s Disease: A Network Meta-Analysis

**DOI:** 10.3390/brainsci12111496

**Published:** 2022-11-04

**Authors:** Yuxia Zhu, Sichen Li, Hongyu Lai, Lijuan Mo, Changhong Tan, Xi Liu, Fen Deng, Lifen Chen

**Affiliations:** Department of Neurology, The Second Affiliated Hospital of Chongqing Medical University, Chongqing 400010, China

**Keywords:** Parkinson’s disease, verbal fluency, letter fluency, semantic fluency, drug therapy, network meta-analysis

## Abstract

Verbal fluency impairment is common in patients with Parkinson’s disease (PD), but the effect of drugs on verbal fluency in PD patients has not been comprehensively evaluated. We conducted a network meta-analysis based on four online databases to compare the effect of drugs on verbal fluency in PD patients. This study was performed and reported according to PRISMA-NMA guidelines. In total, 6 out of 3707 articles (three RCTS and three cross-sectional studies) covering eight drug regimens were included (five for letter fluency, five for semantic fluency). In terms of letter fluency, the ranking of the overall efficacy of included drug regimens was: levodopa, levodopa combined with pramipexole, rotigotine, cabergoline, pramipexole, pergolide, but no drug regimen presented a significant advantage over the others. In terms of semantic fluency, the ranking of the overall efficacy of included drug regimens was: rotigotine, levodopa, cabergoline, pergolide, pramipexole, among which, levodopa alone (SMD = 0.93, 95%CI: 0.28–1.59) and rotigotine alone (SMD = 1.18, 95%CI: 0.28–2.09) were statistically superior to pramipexole, while no significant difference was identified between all the other drug regimens. Levodopa and rotigotine seem to be more appropriate choices for PD patients with verbal fluency impairment. Further study is needed to illustrate the efficacy of drugs on verbal fluency in PD patients.

## 1. Introduction

Communication impairment is a common symptom affecting 90% of Parkinson’s disease (PD) patients; it may be induced by either motor speech control dysfunction or cognitive-linguistic dysfunction [1,2] and may lead to occupational function impairment, social isolation, and depression and affects the quality of life severely [1,2,3]. However, communication impairment in PD is relatively overlooked in comparison with other symptoms [1]. Communication impairments, including verbal fluency impairment, speech-acoustic changes, impairment in production and processing of grammar and syntax, dysfunction of action word use, and pauses [1], may onset early in the course or in the later stage of PD [4]. Among these communication impairments, verbal fluency impairment is related to cognitive or linguistic dysfunction [1] and is the most prominent part of cognitive impairment in PD patients, although it seems to be an uncommon chief complaint of PD patients [5].

Verbal fluency reflects the ability to spontaneously produce words under restricted search conditions [6], which includes three types, namely, letter fluency, semantic/categorical fluency, and motor/action fluency [7,8]. Verbal fluency has been widely measured to assess cognitive function and language content after nervous system impairment [9]. More specifically, letter fluency tests request subjects to produce words in mind with a restricted initial letter (such as “F”, “A”, “S”, and so on) [6], semantic fluency tests request subjects to produce words (nouns) in mind with a restricted category (such as animals, vegetables, fruits, and so on) [6], while motor fluency tests requests subjects to produce words (verbs) in mind [6]. All three types of tests request the subjects to speak out the words that they produce in mind but do not request the subjects to pronounce them clearly. Therefore, all three types of verbal fluency reflect expressive modality of language since they do not request subjects to receive language by reading or hearing. They are not associated with articulation, either, because they do not request subjects to pronounce clearly.

Based on previous neuroanatomical findings, letter fluency is related to frontal lobe function and involves executive function [7]. It has been reported that PD-associated loss of dopaminergic neurons in substantia nigra and subsequent dopamine depletion in nigrostriatal projection may lead to functional damage in subcorticofrontal circuits and, thus, impair executive processes relying on this circuit [10]. Semantic fluency is related to temporal lobe function and involves language function [7]. Interestingly, temporal lobe function is not severely impaired in PD patients without cognitive impairment or dementia, and it does not depend on the dopaminergic system, either [11]; therefore, the mechanism of semantic fluency impairment in PD remains unclear. Motor fluency involves the function of motor and premotor areas [7]. It has been proven that dopamine depletion selectively impairs the procession of verbs in PD patients [7], and PD patients suffer from greater difficulty in naming actions with a high degree of motor content than actions with a low degree of motor content [7]. Some researchers concluded that, in PD patients, deficits in language formulation might be due to impairment of basal ganglia connections to the cortex [5]; however, the damage in such connection could not explain semantic fluency impairment in PD.

Previous studies have found that verbal fluency in PD patients deteriorates with the disease progression and is linearly correlated with the disease stage [12], and it is also impacted by depression, global cognitive ability, and executive dysfunction [12]. Among all types of verbal fluency, semantic fluency is slightly more damaged than letter fluency in PD [10,13,14,15]. Additionally, semantic fluency has been found to be a useful predictor of cognitive decline in PD patients [16,17], indicating that semantic fluency is impaired early in the process of cognitive impairment in PD patients. Interestingly, it is also reported that motor fluency impairment may be particularly sensitive to PD-related dementia (PDD) and may be an early indicator of conversion from PD to PDD [8].

The current therapeutic strategies for PD include drug therapy [12,16,17,18,19,20,21,22], non-invasive brain stimulation [23], aerobic exercise therapy [24], and deep brain stimulation [5,25,26]. Among these therapies, deep brain stimulation of the subthalamic nucleus or the internal segment of the globus pallidus is reported to exacerbate patients’ verbal fluency impairment [5,26,27]. As the most commonly used therapy for PD, drugs present complex effects on verbal fluency in patients; for example, levodopa improves the verbal fluency of PD patients [14], while pramipexole exacerbates their verbal fluency impairment [19]. However, there is still a lack of comprehensive and systematic evaluation and comparison of the effect of drugs on verbal fluency in PD patients, and further research is necessary for a better quality of life for PD patients and a better clinical decision.

This network meta-analysis (NMA) aimed to compare the effect of drugs on verbal fluency in PD patients and to assist clinical decisions.

## 2. Methods

This study was performed and reported according to the Preferred Reporting Items for Systematic Reviews and Meta-Analyses for Network Meta-Analyses (PRISMA-NMA) guidelines (Supplementary Table S1). This study has not been pre-registered.

### 2.1. Search Strategy

We searched literature published in English or in Chinese in PubMed, Embase, Cochrane Library and Chinese National Knowledge Infrastructure (CNKI) database from the date of their inception to 10 February 2021, using the following search strategy: (((Parkinson *) OR Paralysis Agitans) AND drug therapy) AND (((((dysarthria OR speech) OR voice) OR fluency) OR intelligibility) OR dysphonia).

### 2.2. Inclusion and Exclusion Criteria

The titles and abstracts of studies were initially screened by two investigators separately (Y.Z., S.L.). Arguments between the two investigators were solved by a third investigator (X.L.). The full text of the article was further evaluated after initial screening. Inclusion criteria were as follows: (1) the included studies must be randomized controlled trials, cohort studies, case-control studies, or cross-sectional studies; (2) the subjects should be PD patients with verbal fluency impairment; (3) studies should investigate effects of drug therapy on verbal fluency in PD; (4) the included studies must provide clear diagnostic criteria for PD and assessment criteria for verbal fluency; (5) indicators such as standardized mean difference (SMD) must be provided to reflect the drug effect or could be calculated based on the data provided in the literature.

Exclusion criteria were as follows: (1) studies without appropriate control group; (2) studies with subjects who had undergone neurosurgical operations (such as DBS); (3) studies provided no medication regimen; (4) studies with subjects having psychosis or dementia.

### 2.3. Assessment of Quality of Literature

The quality of all included studies was independently assessed by two researchers (Y.Z., S.L.). The Cochrane Handbook for Systematic Reviews of Interventions (version 6.2, 2021) [28] was used for the assessment of RCT studies. Newcastle-Ottawa Scale was used for the assessment of cohort studies and case-control studies [29]. An assessment form formulated by Agency for Healthcare Research and Quality (AHRQ) was used for the assessment of cross-sectional studies [30]. A third party was consulted to resolve disagreement between the two researchers.

### 2.4. Data Selection

The following data were collected from each included literature: (1) basic characteristics of the literature (last name of the first author, publication year, and the country/region in which the study was performed), (2) study design (research types, diagnostic criteria of PD, drug intervention plans, and the number of participants in groups), (3) characteristics of the participants (mean age and/or age range, sex distribution, and Hoehn-Yahr (H-Y) stage), and (4) evaluation method or criteria of verbal fluency.

### 2.5. Statistical Analysis

In this NMA, SMD with a 95% confidence interval (CI) was used as the effect analysis statistic. SMDs were calculated as follows: The SMDs were generated from the median of the posterior distribution, according to the algorithms in the Cochrane Handbook [28]. The 2.5th and 97.5th percentiles of the posterior distribution were considered as the lower and upper limit, respectively, of the traditional corresponding 95% CI. Significant differences were identified when the 95% CI did not include 0 for SMD. The heterogeneity of the effect size across the included studies, defined by the differences between direct and indirect effect estimates for the same comparison [31], was tested using the Q test (*p* < 0.05 was considered heterogeneous) and I^2^ (I^2^ > 50% was considered heterogeneous). If there was significant heterogeneity between studies, a random-effects model was used; otherwise, a fixed-effects model was used. Rankings for all evaluated treatments were based on the level of effect according to their posterior probabilities. The surface under the cumulative ranking (SUCRA) is equal to 100% for the best treatment and 0% for the worst treatment [31,32]. All statistical analyses were performed using Stata software (V.16.0, Stata, College Station, TX, USA).

## 3. Results

### 3.1. Search Results and Characteristics of Included Articles

In total, 617 articles from PubMed, 2792 articles from EMBASE, and 298 articles from Cochrane Library were obtained. No relevant article was found in the CNKI database. After excluding duplicates, a total of 3356 articles were obtained for screening. In the screening process, 3195 articles were excluded by screening the titles and abstracts, mainly because of inappropriate study type (e.g., reviews, case reports, letters, editorial, comment, laboratory studies, meta-analysis, and trial involving animals), or irrelevance to drug therapy or verbal fluency impairment. Among the remaining 161 articles, 155 articles were excluded because they did not meet the inclusion criteria or met the exclusion criteria. Additionally, among these studies, one study researched the effect of galanthamine on verbal fluency in patients with PDD [33], one study researched the effect of intranasal insulin administration on letter fluency in PD patients [34], one study researched the effect of statin on verbal fluency in PD patients [21], but data necessary for NMA could not be extracted or calculated from these three studies; therefore, these three studies were excluded.

**Figure 1:**
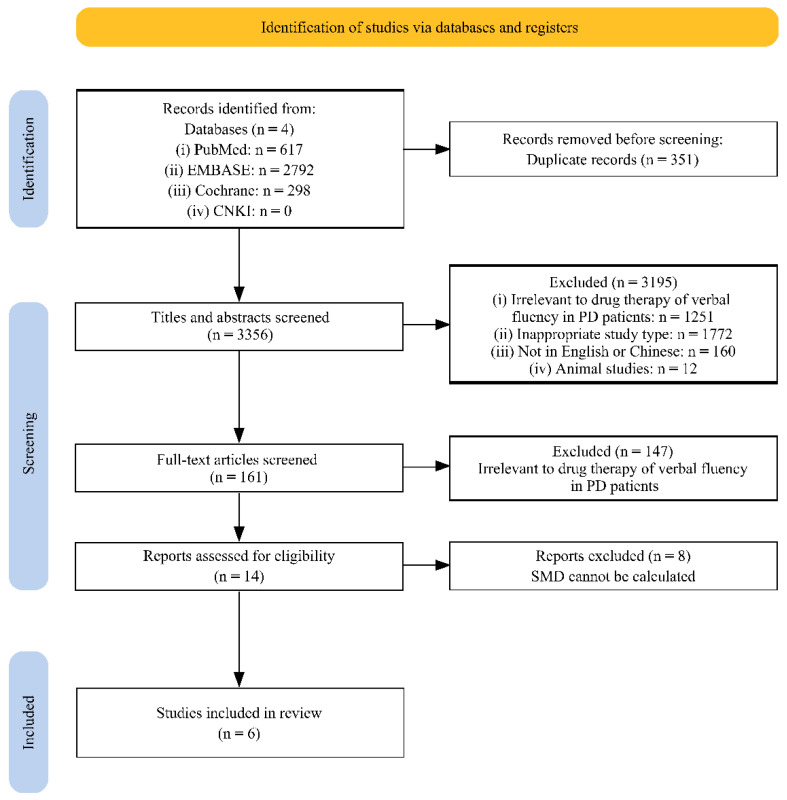
PRISMA flow-chart displaying study selection process. Diagram adapted from Reference [35].

Finally, six articles (three RCTs and three cross-sectional studies), including a total of 198 participants (278 samples due to sequential multiple drug therapy for participants in some studies), were included (Figure 1) [35]. Among the six included articles, five studies investigated letter fluency [18,19,20,22,23], and five studies investigated semantic fluency [14,18,19,20,22]. The basic information of the included studies is shown in Table 1. There was insufficient data for meta-analysis on speech acoustic changes, production and procession of grammar and syntax, action word use, and pauses.

Three studies [14,18,19] presented a moderate risk of bias, and the other three studies presented an unclear risk of bias (Supplementary Tables S2 and S3).

Additionally, to evaluate letter fluency in PD patients, four of the five included studies [18,19,20,23] used the F-A-S test, while one study used the K-A-S test [22], both of which are quite similar. To evaluate semantic fluency in PD patients, all six included studies [14,18,19,20,22] used a category naming test.

### 3.2. Effect of Drug Therapy on Letter Fluency in PD Patients

**Figure 2:**
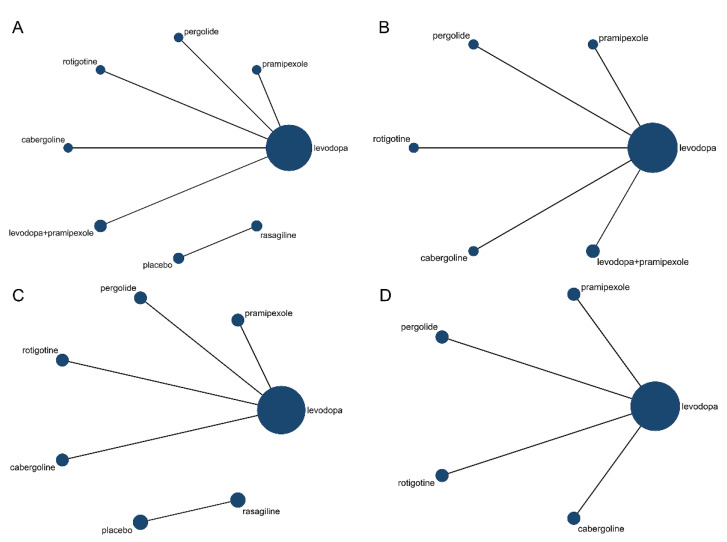
Evidence network diagram of this network meta-analysis. (**A**) Evidence network diagram of letter fluency with rasagiline; (**B**) evidence network diagram of letter fluency without rasagiline; (**C**) evidence network diagram of semantic fluency with rasagiline; (**D**) evidence network diagram of semantic fluency without rasagiline. Because rasagiline did not present connection with other drug regimens, it was excluded from this NMA. Each spot indicates a drug regimen, the size of spot indicates the total number of participants, and each line indicates a comparison between the connected drug regimens.

A total of six trials involving seven drug regimens and placebo were analyzed. Among the six trials, only one trial used rasagiline and placebo and was excluded from the NMA because it could not form a network connection with the other studies (Figure 2A). Finally, five articles involving six drug regimens were included.

**Figure 3:**
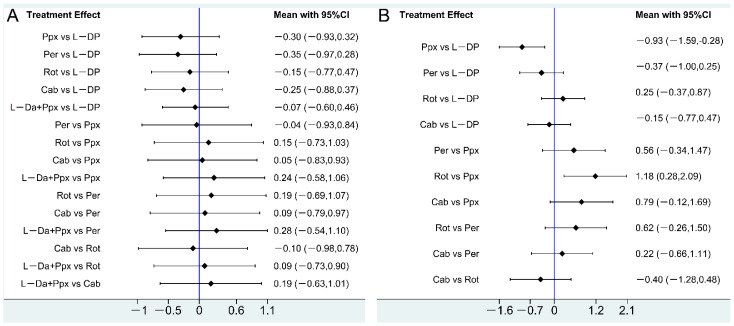
Forest plots of pairwise comparison. (**A**) Forest plots of pairwise comparison of this NMA for all included drugs on letter fluency; (**B**) forest plots of pairwise comparison of this NMA for all included drugs on semantic fluency. L—DP, Levodopa; L—DP + Ppx, Levodopa combined with pramipexole; Per, Pergolide; Ppx, Pramipexole; Rot, Rotigotine; Cab, Cabergoline.

**Figure 4:**
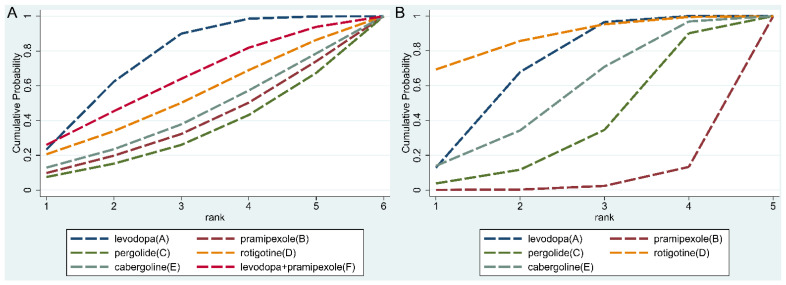
Surface under the cumulative ranking curve (SUCRA). (**A**) SUCRA of all included drugs on letter fluency; (**B**) SUCRA of all included drugs on semantic fluency.

The structure of the network formed by drug regimens is shown in Figure 2B. In the included studies, levodopa, pergolide, rotigotine, and cabergoline were reported to be effective in improving letter fluency in PD patients, although with controversy. However, pramipexole was reported to exacerbate letter fluency in PD patients [19]. This NMA showed that no drug regimen presented a significant advantage in letter fluency in PD patients over the others (Figure 3A). SUCRA (Figure 4A) showed that, although no drug regimen had a significant advantage in letter fluency over the other regimens, levodopa alone (SUCRA = 75.2%), and levodopa combined with pramipexole (SUCRA = 63.0%) presented a trend that they may have stronger improving effect on letter fluency impairment in PD patients, followed by rotigotine (SUCRA = 51.9%), cabergoline (SUCRA = 41.6%), pramipexole (SUCRA = 36.3%), and pergolide (SUCRA = 32%). Additionally, rasagiline did not present a significant improvement in letter fluency in PD patients compared with placebo (*p* = 0.156) [22]. More detailed data are shown in Supplementary Tables S4 and S5.

**Figure 5:**
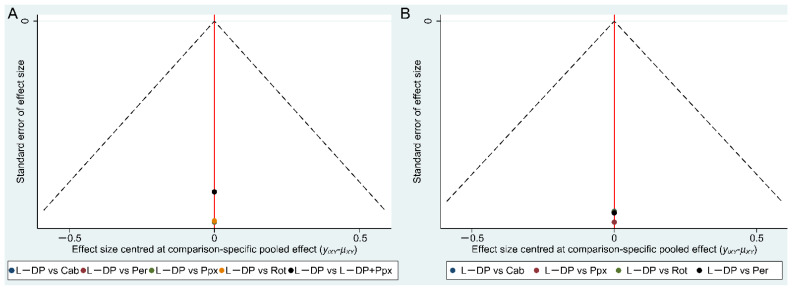
Funnel plots of the risk of publication bias. (**A**) Funnel plots of the risk of publication bias for the included literature on letter fluency; (**B**) funnel plots of the risk of publication bias for the included literature on semantic fluency.

The funnel plot (Figure 5A) indicated that no publication bias exists.

### 3.3. Effect of Drug Therapy on Semantic Fluency in PD Patients

A total of six trials involving six drug regimens and placebo were analyzed. Among the six trials, only one trial compared rasagiline and placebo and was excluded from the NMA because it could not form a network connection with the other studies (Figure 2C). Finally, five articles involving five drug regimens were included.

The structure of the network formed by drug interventions is shown in Figure 2D. In the included studies, levodopa, pergolide, rotigotine, and cabergoline were reported to be effective in improving semantic fluency in PD patients. However, pramipexole was reported to exacerbate semantic fluency in PD patients [19]. NMA showed that levodopa alone (SMD = 0.93, 95%CI: 0.28–1.59) and rotigotine alone (SMD = 1.18, 95%CI: 0.28–2.09) were statistically superior to pramipexole alone, while no significant difference was identified between all the other drug regimens (Figure 3B). SUCRA (Figure 4B) showed that among all drug regimens, rotigotine alone (SUCRA = 87.0%) and levodopa alone (SUCRA = 69.2%) seem to present relatively stronger effect on improving semantic fluency impairment in PD patients, followed by cabergoline (SUCRA = 54.2%), pergolide (SUCRA = 35.7%) and pramipexole (SUCRA = 3.9%). Additionally, rasagiline did not present a significant improvement effect on semantic fluency impairment in PD patients compared with placebo (*p* = 0.06) [22]. More detailed data are shown in Supplementary Tables S6 and S7.

The funnel plot (Figure 5B) indicated no publication bias exists.

## 4. Discussion

This NMA found that: (1) levodopa, pergolide, rotigotine, and cabergoline showed similar improving effects on verbal fluency (both letter fluency and semantic fluency) impairment in PD patients, and no drug presented a significant advantage over the others, which is consistent with previous reports [14,18,19,20]; (2) in this NMA, pramipexole showed a weaker effect on semantic fluency compared with levodopa and rotigotine, while pramipexole had a similar effect [19] on semantic fluency compared with other drug regimens; (3) levodopa and rotigotine presented relatively higher ranking in both letter fluency and semantic fluency compared with other drug regimens. Additionally, with insufficient data for NMA, rasagiline did not present a stronger effect on both letter fluency and semantic fluency compared with placebo in previous studies [22].

Letter fluency, based on phonemic fluency, is considered to depend on frontal lobe function and reflect executive function [36,37,38], while semantic fluency, based on category fluency, is thought to depend on temporal lobe function and reflect memory function [36,37,38]. Interestingly, the mechanism of memory (including working memory) and executive function were reported to be critically dependent on D1 and D2 dopamine receptors [39,40]. Meanwhile, D3, D4, and D5 dopamine receptors were also reported to have a slight regulatory influence on some aspects of cognitive function [41,42,43]. These previous findings suggest a possible mechanism that levodopa, cabergoline, and pergolide improve letter fluency and semantic fluency in PD patients [18,19,20], possibly by activating D1 and D2 receptors [44,45]. Therefore, the unbalanced effect of pramipexole on dopamine receptors may possibly explain its exacerbative effect on verbal fluency in PD patients [18,23]. However, rotigotine, which has a much weaker effect on D1 dopamine receptor compared with D2, D3, D4, and D5 receptors [44], presents an improving effect on both letter fluency and semantic fluency [20], suggesting that dopamine receptors other than the D1 receptor may play a role in verbal fluency impairment in PD patients [46]. These findings suggest complex roles and interactions of dopamine receptors in the mechanism of verbal fluency impairment in PD.

More interestingly, previous studies have also found that long-term use of clozapine, which has a strong blockade of the D1 dopamine receptor, a weak blockade of the D2 dopamine receptor, and an anticholinergic effect, improves verbal fluency in patients with schizophrenia [47], while, risperidone, the antagonist of the D2 dopamine receptor, presents similar improvement in verbal fluency in patients with schizophrenia compared with clozapine [47]. It is possible that a balance between activation and inhibition of dopamine receptors may play a role in verbal fluency.

Although this NMA did not show significant differences between drug regimens in letter fluency impairment in PD patients, levodopa and rotigotine ranked first and second in improving letter fluency impairment in PD patients in SUCRA, respectively. Most of the included drugs, except pergolide, ranked higher than pramipexole. In the SUCRA ranking of drug effects on semantic fluency, rotigotine was ranked first, while levodopa was ranked second. These findings suggest that levodopa and rotigotine could be considered appropriate choices for PD patients with verbal fluency impairment. Clinically, cabergoline and pergolide are no longer recommended for the treatment of PD due to side effects such as pleural pulmonary fibrosis and fibrotic valvular heart disease [44]. Notably, this NMA is only a statistical comparison between drugs; whether the drugs are clinically effective remains to be further observed.

To the best of our knowledge, there is no similar meta-analysis or NMA investigating drug effect on verbal fluency in PD patients comprehensively. Regarding each specific drug, our NMA presented a similar effect on verbal fluency in PD patients compared with previous studies [14,18,20,22,23]. However, there was one previous study that reported that the letter fluency of PD patients deteriorated after levodopa administration [23]. Compared with the other studies reporting the improving effect of levodopa on letter fluency in PD patients, we found that PD patients in this study [23] had the longest period of treatment for 6 months. It is possible that the duration of levodopa administration may affect its effect on letter fluency [45,48], and it may also be attributed to the progress of the disease and the effect of long time intervals on verbal fluency tests. There was also one previous study reporting the deteriorative effect of pramipexole on letter fluency [19], while this NMA only found a deteriorative trend of pramipexole treatment on letter fluency without statistical significance, possibly due to multiple indirect comparisons in NMA. Therefore, in patients receiving pramipexole, their verbal fluency should be tensely observed, and further research is needed.

Additionally, in the searched literature, some non-anti-parkinsonian drugs seem to influence verbal fluency, too; however, data on these drugs are insufficient for NMA. Specifically, galanthamine has been reported to improve verbal fluency in patients with PDD [33]. Intranasal insulin administration has been found to improve letter fluency in PD patients [34]. Statin users were found to perform better in verbal fluency than non-statin users, and presented a lower rate of verbal fluency decline [21]. The use of non-anti-parkinsonian drugs may be new therapeutic approaches to alleviate verbal fluency impairment in PD patients if further researched.

Notably, none of the six included studies analyzed the effect of drugs on motor fluency impairment in PD. This lack of study may be attributed to the fact that we excluded studies involving PDD patients because motor fluency impairment has been proven to be particularly sensitive to PD-associated dementia and may be an early indicator of the conversion from PD to PDD [8]. Moreover, motor fluency is less noticed in the clinic, and it is also more difficult for PD patients to complete motor fluency tests [8].

Several limitations exist for this NMA: (1) limited number of studies with limited participants included in this NMA affected the reliability and stability of the results; (2) only studies published in English are included, which may cause bias; (3) lack of direct comparison between the included drug regimens resulted in a failure that no closed loop was formed in evidence network diagram, so the comparisons between drugs in this NMA were indirect; (4) the tests used to assess letter fluency among the included studies were much similar but not the same; (5) the duration of drug administration in the included studies was different; (6) all the included studies in this NMA excluded PDD patients; therefore, this NMA could not draw conclusion on the effect of cognitive function on verbal fluency in PD patients, especially considering that cognitive function status may affect verbal fluency [8]; (7) this NMA did not analyze the effect of language differences on the results of verbal fluency tests, which is known to affect both letter fluency tests and semantic fluency tests [49].

## 5. Conclusions

Levodopa, rotigotine, carbergoline, pergolide, pramipexole, and levodopa combined with pramipexole showed a similar effect on letter fluency, while levodopa, rotigotine presented a stronger effect on semantic fluency compared with pramipexole. It is reasonable to select drug regimens for PD patients based on their motor and non-motor symptoms, as well as the tolerance of drugs, adverse reactions, and individual conditions. Consequently, levodopa and rotigotine seem to be more appropriate choices for PD patients with verbal fluency impairment. More studies of higher quality and larger sample sizes are needed to further illustrate the effect of drugs on verbal fluency impairment in PD patients.

## Figures and Tables

**Table 1:** Characteristics of included studies.

Study	Country	Study Design	Type of Interventions	Sample Size	Hoehn and Yahr Scale	Gender (Female/Male)	Age (Year)	Duration (Year)	Outcome Evaluation Index	Period of Treatment	Change of Scores after Drug Administration (Mean ± SD)
Relja [23] 2006	Croatia	RCT	levodopa	25	2.3 ± 1	10/15	63.0 ± 12.7	5.4 ± 3.1	Letter fluency	6 months	−0.6 ± 7.95
			levodopa & pramipexole	30	2.0 ± 0.8	13/17	61.7 ± 14.2	4.6 ± 4.8	Letter fluency	6 months	−1.2 ± 9.7
Gotham [14] 1988	UK	cross-sectional	levodopa	15	NA	NA	64.4 ± 5.9	9.9 ± NA	Semantic fluency	On-Off	1.72 ± 3.34
Brusa [18] 2005	Italy	cross-sectional	levodopa	20	≤2.5	7/13	58 ± 7.83	2.6 ± 1.8	Letter fluency Semantic fluency	16 weeks	6.25 ± 10.13 2.97 ± 5.6
			pergolide	20	≤2.5	7/13	58 ± 7.83	2.6 ± 1.8	Letter fluency Semantic fluency	16 weeks	2.63 ± 10.43 1.24 ± 3.22
Brusa [19] 2003	Italy	cross-sectional	levodopa	20	≤2.5	7/13	57 ± 9.32	2.5 ± 1.3	Letter fluency Semantic fluency	4 months	0.94 ± 9.22 2.33 ± 2.57
			pramipexole	20	≤2.5	7/13	57 ± 9.32	2.5 ± 1.3	Letter fluency Semantic fluency	4 months	−1.93 ± 9.38 −0.73 ± 3.75
Brusa [20] 2013	Italy	RCT	levodopa	20	≤2.5	NA	56 ± 5.63	2.3 ± 1.4	Letter fluency Semantic fluency	3 months	6.71 ± 11.92 0.53 ± 5.85
			rotigotine	20	≤2.5	NA	56 ± 5.63	2.3 ± 1.4	Letter fluency Semantic fluency	3 months	5 ± 10.08 1.98 ± 5.51
Brusa [20] 2013	Italy	RCT	levodopa	20	≤2.5	NA	57 ± 2.13	3.1 ± 0.5	Letter fluency Semantic fluency	3 months	3.11 ± 9.69 0.79 ± 4.31
			cabergoline	20	≤2.5	NA	57 ± 2.13	3.1 ± 0.5	Letter fluency Semantic fluency	3 months	0.69 ± 9.01 0.22 ± 3.19
Hanagasi [22] 2011	Turkey	RCT	rasagiline	23	2.00 ± 0.69	6/17	65.17 ± 9.5	4.09 ± 2.54	Letter fluency Semantic fluency	12 weeks	3.14 ± 6.78 1.45 ± 4.44
			placebo	25	1.64 ± 0.60	9/16	67.56 ± 10.13	3.96 ± 2.26	Letter fluency Semantic fluency	12 weeks	0.52 ± 5.66 −0.72 ± 5.16

NA, not applicable; SD, Standard Deviation; RCT, Randomized Control Trial; UK, United Kingdom; On–Off, Time of Drug Onset.

## Data Availability

No new data were created or analyzed in this study. Data sharing is not applicable to this article.

## Supplementary Materials

The following supporting information can be downloaded at: https://www.mdpi.com/article/10.3390/brainsci12111496/s1. Supplementary Table S1: PRISMA-NMA Checklist; Supplementary Table S2: Quality Assessment of Cross-sectional Studies; Supplementary Table S3: Quality Assessment of Randomized Control Trial Studies; Supplementary Table S4: Primary Outcomes of Letter Fluency Improvements; Supplementary Table S5: Surface Under the Cumulative Ranking Curve (SUCRA); Supplementary Table S6: Primary Outcomes of Semantic Fluency Improvements; Supplementary Table S7: Surface Under the Cumulative Ranking Curve (SUCRA).
