# Supplementary material for: Effects of Anti-Parkinsonian Drugs on Verbal Fluency in Patients with Parkinson’s Disease: A Network Meta-Analysis
Source: Brain Sci. 2022 Nov 4;12(11):1496. doi: 10.3390/brainsci12111496 (PMC9688928; doi:10.3390/brainsci12111496)
Supplement: Supplementary file 1 [file brainsci-12-01496-s001.zip › 22-11-3 Supplementary Table.pdf]

Supplementary Table S2. Quality Assessment of Cross-sectional Studies.

Study	Study Design	Define the source	List inclusion and exclusion criteria	Indicate time period	Population-based	Evaluators were masked to other aspects	Assessments for quality assurance	Explain exclusions	Describe confounding	Explain missing data	Summarize response rates and completeness	Clarify follow-up	Total score
Gotham [14] 1988	cross-sectional	Yes	Yes	No	No	Unclear	Unclear	Unclear	Yes	Unclear	Yes	Unclear	4
Brusa [18] 2005	cross-sectional	Yes	Yes	Yes	No	Unclear	Unclear	Unclear	Yes	Unclear	Yes	Yes	6
Brusa [19] 2003	cross-sectional	Yes	Yes	Yes	No	Unclear	Unclear	Unclear	Yes	Unclear	Yes	Yes	6

Supplementary Table S3. Quality Assessment of Randomized Control Trial Studies.

Study	Study Design	Random sequence generation (selection bias)	Allocation concealment (selection bias)	Blinding of participants and personnel (performance bias)	Blinding of outcome assessment (detection bias)	Incomplete outcome data (attrition bias)	Selective reporting (reporting bias)	Other bias	Total risk
Relja [23] 2006	RCT	Unclear	Unclear	Low	Unclear	Low	Unclear	Low	Unclear
Brusa [20] 2013	RCT	Unclear	Unclear	Low	Low	Low	Unclear	Low	Unclear
Hanagasi [22] 2011	RCT	Unclear	Low	Low	Low	Low	Unclear	Low	Unclear

RCT, randomized control trial.

Supplementary Table S4. Primary Outcomes of Letter Fluency Improvements.

Levodopa	-0.07 (-0.60,0.46)	-0.15 (-0.77,0.47)	-0.25 (-0.88,0.37)	-0.30 (-0.93,0.32)	-0.35 (-0.97,0.28)
0.07 (-0.46,0.60)	Levodopa + Pramipexole	-0.09 (-0.90,0.73)	-0.19 (-1.01,0.63)	-0.24 (-1.06,0.58)	-0.28 (-1.10,0.54)
0.15 (-0.47,0.77)	0.09 (-0.73,0.90)	Rotigotine	-0.10 (-0.98,0.78)	-0.15 (-1.03,0.73)	-0.19 (-1.07,0.69)
0.25 (-0.37,0.88)	0.19 (-0.63,1.01)	0.10 (-0.78,0.98)	Cabergoline	-0.05 (-0.93,0.83)	-0.09 (-0.97,0.79)
0.30 (-0.32,0.93)	0.24 (-0.58,1.06)	0.15 (-0.73,1.03)	0.05 (-0.83,0.93)	Pramipexole	-0.04 (-0.93,0.84)
0.35 (-0.28,0.97)	0.28 (-0.54,1.10)	0.19 (-0.69,1.07)	0.09 (-0.79,0.97)	0.04 (-0.84,0.93)	Pergolide

Supplementary Table S5. Surface Under the Cumulative Ranking Curve (SUCRA) of Letter Fluency.

Treatment	SUCRA	PrBest	MeanRank
Levodopa	75.2	22.4	2.2
Pramipexole	36.3	9.4	4.2
Pergolide	32.0	7.1	4.4
Rotigotine	51.9	21.1	3.4
Cabergoline	41.6	12.6	3.9
Levodopa + Pramipexole	63.0	27.4	2.9

Supplementary Table S6. Primary Outcomes of Semantic Fluency Improvements.

Rotigotine	-0.25 (-0.87,0.37)	-0.40 (-1.28,0.48)	-0.62 (-1.50,0.26)	**-1.18 (-2.09,-0.28)**
0.25 (-0.37,0.87)	Levodopa	-0.15 (-0.77,0.47)	-0.37 (-1.00,0.25)	**-0.93 (-1.59,-0.28)**
0.40 (-0.48,1.28)	0.15 (-0.47,0.77)	Cabergoline	-0.22 (-1.11,0.66)	-0.79 (-1.69,0.12)
0.62 (-0.26,1.50)	0.37 (-0.25,1.00)	0.22 (-0.66,1.11)	Pergolide	-0.56 (-1.47,0.34)
**1.18 (0.28,2.09)**	**0.93 (0.28,1.59)**	0.79 (-0.12,1.69)	0.56 (-0.34,1.47)	Pramipexole

Bold numbers represent the differences are of significances.

Supplementary Table S7: Surface Under the Cumulative Ranking Curve (SUCRA) of Semantic Fluency.

Treatment	SUCRA	PrBest	MeanRank
Levodopa	69.2	13.2	2.2
Pramipexole	3.9	0.0	4.8
Pergolide	35.7	3.9	3.6
Rotigotine	87.0	68.1	1.5
Cabergoline	54.2	14.7	2.8
